# Identification of lncRNA/circRNA-miRNA-mRNA ceRNA Network as Biomarkers for Hepatocellular Carcinoma

**DOI:** 10.3389/fgene.2022.838869

**Published:** 2022-03-21

**Authors:** Shanshan Chen, Yongchao Zhang, Xiaoyan Ding, Wei Li

**Affiliations:** Cancer Center, Beijing Ditan Hospital, Capital Medical University, Beijing, China

**Keywords:** bioinformatics analysis, hepatocellular carcinoma, competing endogenous RNA network, biomarker, diagnosis, prognosis

## Abstract

**Background:** Hepatocellular carcinoma (HCC) accounts for the majority of liver cancer, with the incidence and mortality rates increasing every year. Despite the improvement of clinical management, substantial challenges remain due to its high recurrence rates and short survival period. This study aimed to identify potential diagnostic and prognostic biomarkers in HCC through bioinformatic analysis.

**Methods:** Datasets from GEO and TCGA databases were used for the bioinformatic analysis. Gene Ontology (GO) and Kyoto Encyclopedia of Genes and Genomes (KEGG) enrichment analyses were carried out by WebGestalt website and clusterProfiler package of R. The STRING database and Cytoscape software were used to establish the protein-protein interaction (PPI) network. The GEPIA website was used to perform expression analyses of the genes. The miRDB, miRWalk, and TargetScan were employed to predict miRNAs and the expression levels of the predicted miRNAs were explored *via* OncomiR database. LncRNAs were predicted in the StarBase and LncBase while circRNA prediction was performed by the circBank. ROC curve analysis and Kaplan-Meier (KM) survival analysis were performed to evaluate the diagnostic and prognostic value of the gene expression, respectively.

**Results:** A total of 327 upregulated and 422 downregulated overlapping DEGs were identified between HCC tissues and noncancerous liver tissues. The PPI network was constructed with 89 nodes and 178 edges and eight hub genes were selected to predict upstream miRNAs and ceRNAs. A lncRNA/circRNA-miRNA-mRNA network was successfully constructed based on the ceRNA hypothesis, including five lncRNAs (DLGAP1-AS1, GAS5, LINC00665, TYMSOS, and ZFAS1), six circRNAs (hsa_circ_0003209, hsa_circ_0008128, hsa_circ_0020396, hsa_circ_0030051, hsa_circ_0034049, and hsa_circ_0082333), eight miRNAs (hsa-miR-150-5p, hsa-miR-19b-3p, hsa-miR-23b-3p, hsa-miR-26a-5p, hsa-miR-651-5p, hsa-miR-10a-5p, hsa-miR-214-5p and hsa-miR-486-5p), and five mRNAs (CDC6, GINS1, MCM4, MCM6, and MCM7). The ceRNA network can promote HCC progression *via* cell cycle, DNA replication, and other pathways. Clinical diagnostic and survival analyses demonstrated that the ZFAS1/hsa-miR-150-5p/GINS1 ceRNA regulatory axis had a high diagnostic and prognostic value.

**Conclusion:** These results revealed that cell cycle and DNA replication pathway could be potential pathways to participate in HCC development. The ceRNA network is expected to provide potential biomarkers and therapeutic targets for HCC management, especially the ZFAS1/hsa-miR-150-5p/GINS1 regulatory axis.

## Introduction

As one of the most common malignant tumors of digestive system, hepatocellular carcinoma (HCC) is characterized by diverse etiology, high incidence, and poor prognosis. According to statistics, both its incidence and mortality rates are the highest in Asia (Singal et al., 2020). Due to the lack of obvious symptoms, the majority of HCC patients are diagnosed at advanced stages, with limited treatment options. Despite progress has been made in diagnosis and therapy during the last decades, the recurrence and metastasis rates remain high (Ghouri et al., 2017). To confront the current situation, it is crucial to develop new strategies for screening and monitoring of HCC. Alpha-fetoprotein (AFP) is currently the most widely used biomarker for HCC diagnosis, but its sensitivity and specificity are still not satisfactory (Daniele et al., 2004). In recent years, the development of various sequencing platforms and bioinformatics technologies has facilitated the identification of many novel biomarkers. Public databases have provided us with rich and diverse data resources, and we can further improve our understanding of HCC by integrating data from different sources. Although several biomarkers have been reported relating to the diagnosis and prognosis of HCC, such as alpha-fetoprotein lens culinaris agglutin-3 (AFP-L3), des-*γ*-carboxy prothrombin (DCP), glypican-3 (GPC3), and so on, their practical applications are yet to be evaluated (Piñero et al., 2020). With the vigorous development of bioinformatics, many biomarkers have been identified to be associated with the development of HCC, but few of them have been proven to be of practical use. The number of reliable tumor biomarkers that can be used for the early detection and prognostic assessment is still small in clinical practice. Thus, identification of novel potential biomarkers is warranted, which may contribute to update of diagnostic techniques and improvement of therapeutic efficacy.

It is well known that non-coding RNAs (ncRNAs) account for the vast majority of the human transcriptome, mainly including microRNAs (miRNAs), long non-coding RNAs (lncRNAs), and circular RNAs (circRNAs) (Chan and Tay, 2018). MicroRNA (miRNA) is a class of evolutionarily conserved non-coding RNA (ncRNA) with 18–25 nucleotides in length (Kloosterman and Plasterk, 2006). It participates in a series of physiological and pathological processes *via* mediating the post-transcriptional regulation of target genes (Krol et al., 2010). Aberrant expression of numerous miRNAs has been linked to cancer initiation and progression (Lee and Dutta, 2009). Long non-coding RNA (lncRNA) is known as a type of ncRNA whose length exceeds 200 nucleotides. Studies have revealed that lncRNAs play an important role in cancer development through a variety of mechanisms (Bhan et al., 2017). Circular RNA (circRNA) is a newly discovered ncRNA with a closed-loop structure. Compared with the traditional linear RNA, circRNA is more resistant to RNA exonuclease, without terminal 5′ caps and 3′ polyadenylated tails, and thus more stable (Jeck and Sharpless, 2014). CircRNAs have been confirmed to exert effects on regulating cellular metabolism in cancer (Yu T et al., 2019). In recent years, increasing numbers of researchers have dedicated themselves to exploring the biological functions of ncRNAs. Various computational methods have also been developed for the prediction of potential associations between ncRNAs and disease, which is of critical importance for the identification of biomarkers (Lan et al., 2020; Chen et al., 2021).

The competing endogenous RNA (ceRNA) hypothesis was first put forward by Salmena et al., in 2011 (Salmena et al., 2011). CeRNA is a class of ncRNA that can competitively bind shared miRNAs and cross-regulate each other at the post-transcription level. In the cytoplasm, lncRNA and circRNA can serve as miRNA sponges by common miRNA response elements (MREs) and indirectly regulate the downstream target genes (Taulli et al., 2013; Yao et al., 2019). This ceRNA-based regulatory mechanism has been discovered in multiple cancers. For example, lncRNA HOTAIR regulated HER2 expression through competition for miR-331-3p, thereby facilitating tumor development (Liu et al., 2014). LncRNA H19 was reported to exerted oncogenic functions in gallbladder cancer *via* modulating miR-342-3p and FOXM1 (Wang et al., 2016). In addition, circRNA ciRS-7 could act as the “super sponge” of microRNA-7 (miR-7) and inhibits the activity of miR-7 (Peng et al., 2015). Accumulating evidence has indicated that ceRNA regulation network serves a role in biological processes of HCC development, such as proliferation, metastasis, epithelial to mesenchymal transition (EMT), and chemotherapy resistance (Wu et al., 2018; Liu Z et al., 2019; Huang et al., 2020; Song et al., 2020).

**FIGURE 1:**
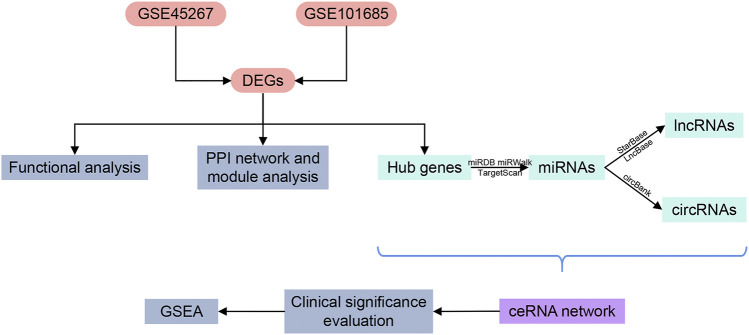
Flowchart of the research design. DEGs, differentially expressed genes; PPI, protein-protein interaction; miRNAs, microRNAs; lncRNAs, long non-coding RNAs; circRNAs, circular RNAs; ceRNA, competing endogenous RNA; GSEA, gene set enrichment analysis.

The ceRNA network provides new perspectives for improving diagnosis and treatment for HCC. Even though several ceRNAs have been found associated with HCC progression (Bai et al., 2019; Guo et al., 2019; Wang et al., 2019; Yu J et al., 2019), our current understanding of ceRNA regulatory network in HCC is still very limited. Further exploration is needed to unravel unknown functions and mechanisms of related ceRNA. Sequencing data used in this study were collected from public databases. Starting from the differentially expressed messenger RNAs (mRNAs), we inversely predicted the targeted miRNAs and their relevant lncRNAs and circRNAs, and then constructed a comprehensive ceRNA network. Bioinformatics tools were applied to analyze and discuss the crucial pathways as well as the diagnostic performance and prognostic value of the key genes. LncRNAs, circRNAs, miRNAs and targeted mRNAs engaged in the ceRNA network may become potential diagnostic biomarkers and therapeutic targets for HCC. The research process is shown in Figure 1.

## Materials and Methods

### Screening of Differentially Expressed Genes

Gene Expression Omnibus (GEO) database^1^ was searched to obtain appropriate gene expression datasets. The GSE45267 dataset (containing 48 HCC samples and 39 non-cancerous samples) and the GSE101685 dataset (containing 24 HCC samples and eight non-cancerous samples) were selected. Differentially expressed genes (DEGs) between HCC tissue and normal liver tissue were identified *via* GEO2R online tools (Barrett et al., 2013). The adjusted *p*-value < 0.01 and the value of log-fold change |logFC| ≥ 1 were set as DEGs cutoff criteria. The visualization of the DEGs was shown on the heat maps and volcano plots, performed by ComplexHeatmap (Gu et al., 2016) and ggplot2 packages, respectively. The VennDiagram (Chen and Boutros, 2011) package of R was used to screen the common DEGs and construct the Venn diagram.

### Functional Enrichment Analysis

WEB-based GEne SeT AnaLysis Toolkit (WebGestalt)^2^ (Liao et al., 2019) is a powerful online tool for functional enrichment analysis. Gene Ontology (GO) and Kyoto Encyclopedia of Genes and Genomes (KEGG) enrichment analyses were carried out by WebGestalt for the upregulated and downregulated DEGs, respectively. GO enrichment analyses include biological process (BP), cellular component (CC), and molecular function (MF). False discovery rate (FDR) < 0.05 was considered statistically significant. The ggplot2 package of R was adopted to visualize the results of functional enrichment analyses.

### Protein-Protein Interaction Network Construction and Module Analysis

The STRING database^3^ (Szklarczyk et al., 2021) was used to obtain the interaction relationships among DEGs. The minimum required interaction score was set to high confidence (0.7). Then the PPI network of DEGs was constructed by Cytoscape 3.7.2 (Shannon et al., 2003). Molecular Complex Detection (MCODE), a plugin in Cytoscape, was applied to screen significant modules in the network. The advanced options were set as degree cutoff = 2, haircut, node score cutoff = 0.2, k-core = 2, and max depth = 100. Subsequently, the KEGG pathway enrichment analyses of genes in the key modules were performed by the clusterProfiler (Yu et al., 2012) package of R. The *p*-value of less than 0.05 was regarded as significant.

### Identification and Verification of Hub Genes

Another plugin in Cytoscape, cytoHubba (Chin et al., 2014), was used to identify hub genes in the network. The top ten nodes ranked by the MCC algorithm were considered as hub genes. The KEGG pathway enrichment analysis was also performed on the hub genes *via* the clusterProfiler package of R. Gene Expression Profiling Interactive Analysis (GEPIA) database^4^ is an online website that can provide customizable analyses based on TCGA and GTEx data (Tang et al., 2017). This database was used to perform expression analyses of the hub genes. The |Log2FC| cutoff was set to 1, and the *p*-value cutoff was set to 0.01. The KEGG pathway enrichment analysis of hub genes was also conducted by the clusterProfiler package of R.

### Identification of miRNAs

Three databases, miRDB^5^ (Chen and Wang, 2020), miRWalk^6^ (Sticht et al., 2018), and TargetScan^7^ (Agarwal et al., 2015) were applied to predict the upstream miRNAs for the hub genes based on the regulatory associations. The VennDiagram package of R was used to obtain the intersection between the predicted sets, which enhanced the reliability of the final results. Then Cytoscape software was used to construct a miRNA-mRNA network. The expression levels of the predicted miRNAs were explored *via* OncomiR^8^ (Wong et al., 2018) database. *p*-value < 0.05 was considered to be statistically significant.

### Identification of lncRNAs and circRNAs and ceRNA Network Construction

The ceRNA network was constructed based on the interaction relationships among lncRNAs, circRNAs, miRNAs, and mRNAs. To be noted, the ceRNA hypothesis suggests that the expression level of ceRNA should be negatively correlated with miRNA expression and positively correlated with mRNA expression. Therefore, we integrate the predicted relationships and the corresponding expression data to obtain more reliable results. Potential lncRNAs interacted with miRNAs were predicted by the intersection of StarBase^9^ (Li J. H et al., 2014) and LncBase^10^ (Paraskevopoulou et al., 2016) databases. The GEPIA database was then used to obtain the expression data of the targeted lncRNAs and screen out differentially expressed lncRNAs. CircRNA prediction was performed by the circBank^11^ (Liu M et al., 2019) database. The GSE97332 dataset and the GSE164803 dataset were selected to screen the common differentially expressed circRNAs. The identification of final predicted circRNAs was based on the intersection of the predicted group and the differentially expressed group. *p* < 0.05 was considered statistically significant. Ultimately, the lncRNA/circRNA-miRNA-mRNA network was visualized by the ggalluvial package of R and the Cytoscape software.

### Diagnostic and Prognostic Analysis of Key Genes

To assess the clinical significance of the key genes in the ceRNA network, we performed diagnostic and prognostic analyses. The expression profiles of mRNA, miRNA, and lncRNA between HCC samples and normal samples were collected from TCGA^12^ and the expression profiles of circRNA were obtained from the GSE97332 dataset and the GSE164803 dataset. The pROC (Robin et al., 2011) package of R was utilized to assess the diagnostic value of the genes *via* performing the receiver operating characteristic (ROC) curve analysis. The area under the ROC curve (AUC) ≥ 0.7 was considered to indicate good discriminatory performance.

The Kaplan-Meier (KM) survival analysis was performed to evaluate the prognostic value of the gene expression. The GEPIA online website provided functions to analyze the correlation between gene expression of mRNA and lncRNA and survival of HCC patients. The OncoLnc^13^ was employed to explore the relationship of miRNA expression with HCC prognosis. Values of *p* < 0.05 were considered significant.

### Gene Set Enrichment Analysis

Data from TCGA was divided into low and high expression groups according to the median value of core genes with great clinical significance. Gene set enrichment analysis (GSEA) (Subramanian et al., 2005) was conducted by GSEA software to investigate important pathways associated with the selected mRNA. Significant pathways were identified by requiring the false discovery rate (FDR) < 0.05.

## Results

### Identification of Differentially Expressed Genes Between Hepatocellular Carcinoma Tissues and Noncancerous Liver Tissues

**FIGURE 2:**
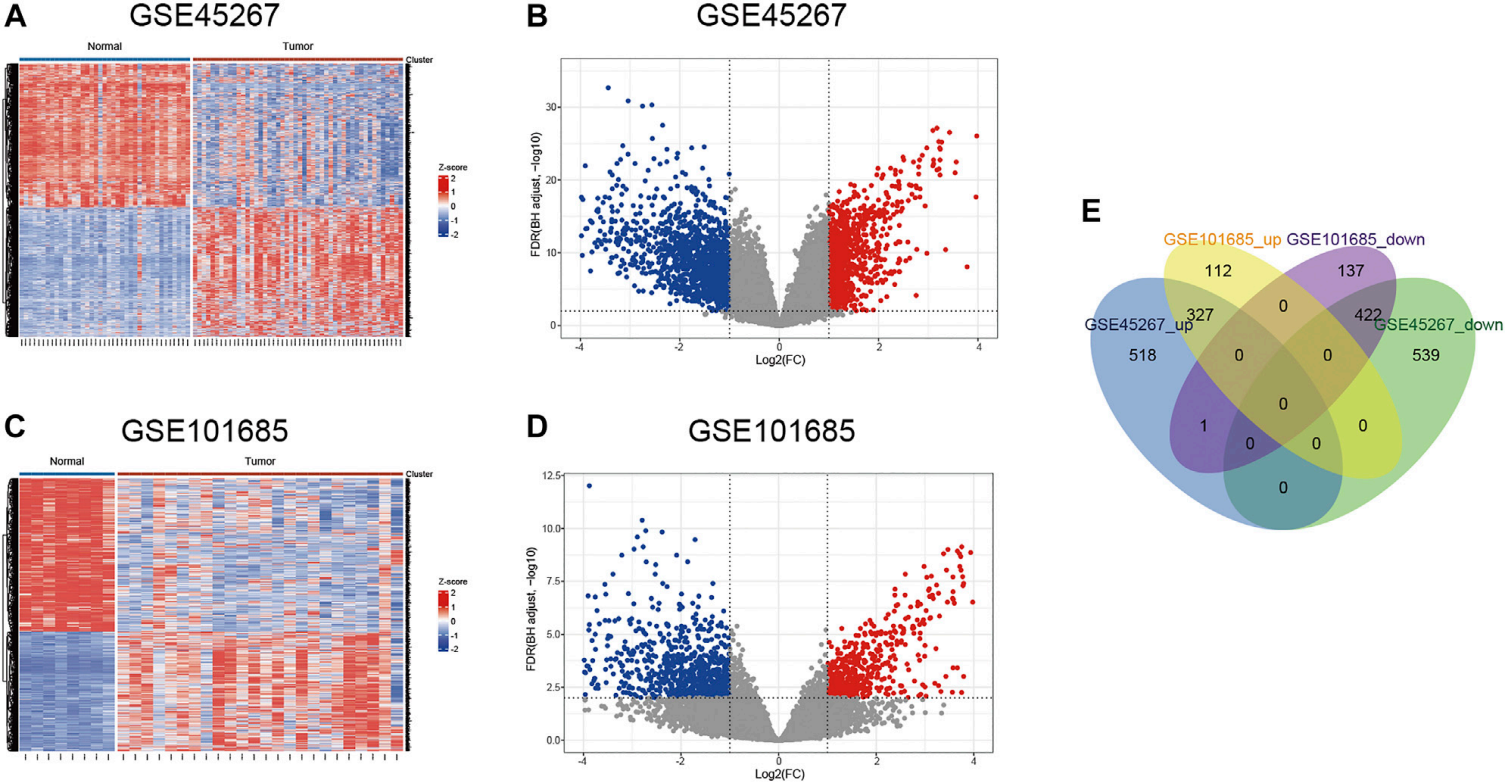
Identification of differentially expressed genes. **(A,B)** Heat map and volcano plot of the GSE45267 dataset. **(C,D)** Heat map and volcano plot of the GSE101685 dataset. **(E)** Venn diagram of the common upregulated and downregulated DEGs. DEGs, differentially expressed genes.

According to the pre-set parameters, 1807 DEGs were screened from the GSE45267 dataset, including 846 upregulated and 961 downregulated genes (Figures 2A,B). There were 999 DEGs obtained from the GSE101685 dataset, containing 439 upregulated and 560 downregulated genes (Figures 2C,D). As shown in Figure 2E, a total of 327 upregulated and 422 downregulated overlapping DEGs were identified between HCC tissues and noncancerous liver tissues.

### Functional Enrichment Analysis of Upregulated and Downregulated Differentially Expressed Genes

**FIGURE 3:**
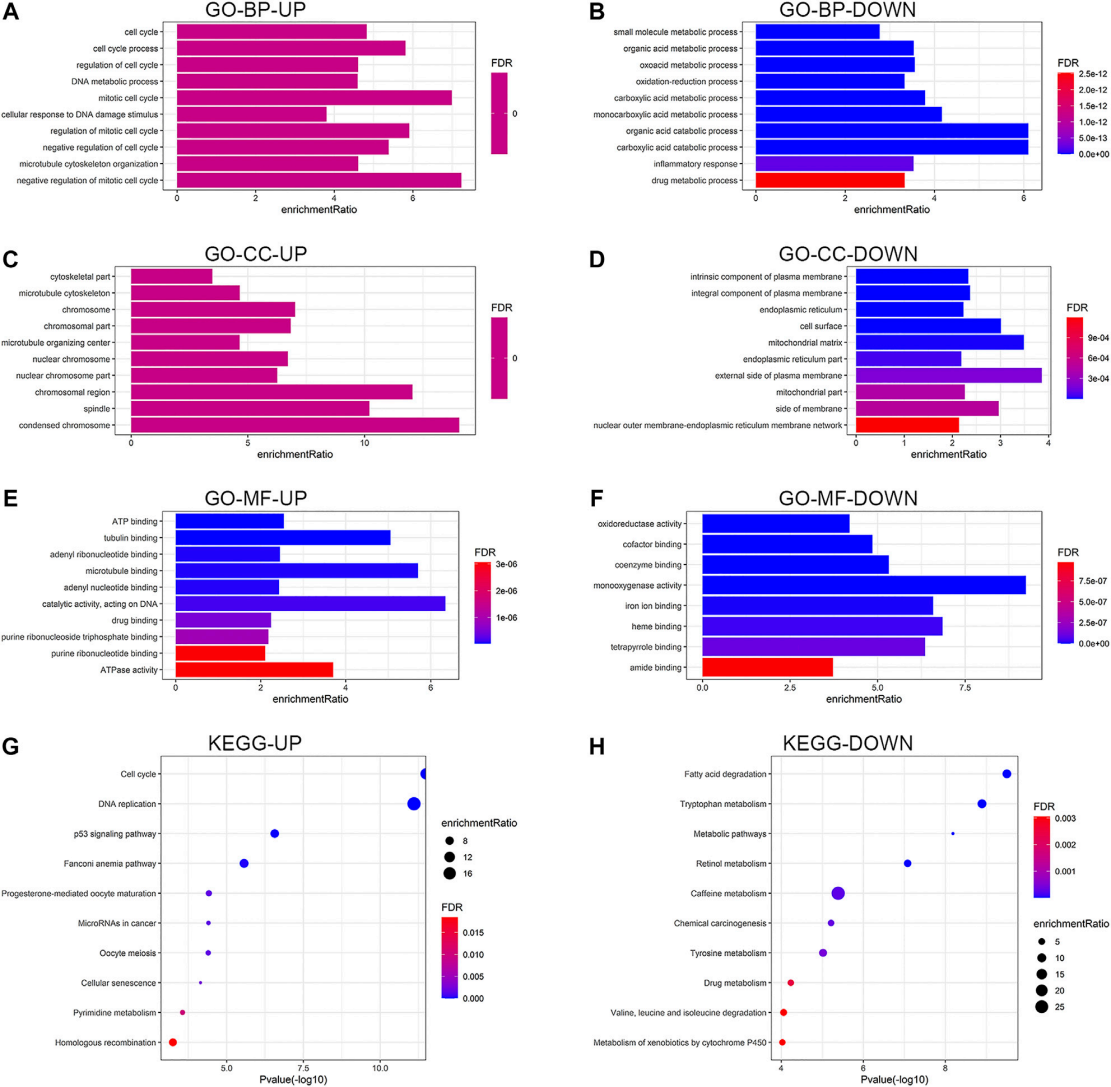
Functional enrichment analysis of the upregulated and downregulated DEGs. Results of GO enrichment analysis of the DEGs, including BP **(A,B)**, CC **(C,D)**, and MF **(E,F)**. **(G,H)** Results of KEGG pathway analysis of the DEGs. DEGs, differentially expressed genes; GO, Gene Ontology; BP, biological process; CC, cellular component, MF, molecular function; KEGG, Kyoto Encyclopedia of Genes and Genomes; UP, upregulated; DOWN, downregulated; FDR, false discovery rate.

Functional enrichment analysis was performed on the common DEGs. Figures 3A–H show the enriched GO functions and KEGG pathways for the upregulated and downregulated DEGs. The upregulated DEGs were mainly enriched in cell cycle, DNA metabolic process, cellular response to DNA damage stimulus, and microtubule cytoskeleton organization in the BP category; condensed chromosome, chromosomal region, and spindle in the cellular component category; catalytic activity, microtubule binding, tubulin binding, and ATPase activity in the molecular function category (Figures 3A,C,E). For the downregulated DEGs, the enriched GO terms were inflammatory response, oxidation-reduction process, and various metabolic processes in the BP category; external side of plasma membrane, mitochondrial matrix, cell surface, and side of membrane in the cellular component category; oxidoreductase activity, monooxygenase activity, heme binding, iron ion binding, and tetrapyrrole binding in the molecular function category (Figures 3B,D,F). As shown in Figures 3G,H, the enriched KEGG pathways for the upregulated DEGs mainly included cell cycle, DNA replication, and p53 signaling pathway, while the downregulated DEGs were highly related to various metabolic pathways. Detailed enrichment results are listed in Supplementary Table S1.

### Protein-Protein Interaction Network, Molecular Complex Detection Analysis and Hub Gene Identification

**FIGURE 4:**
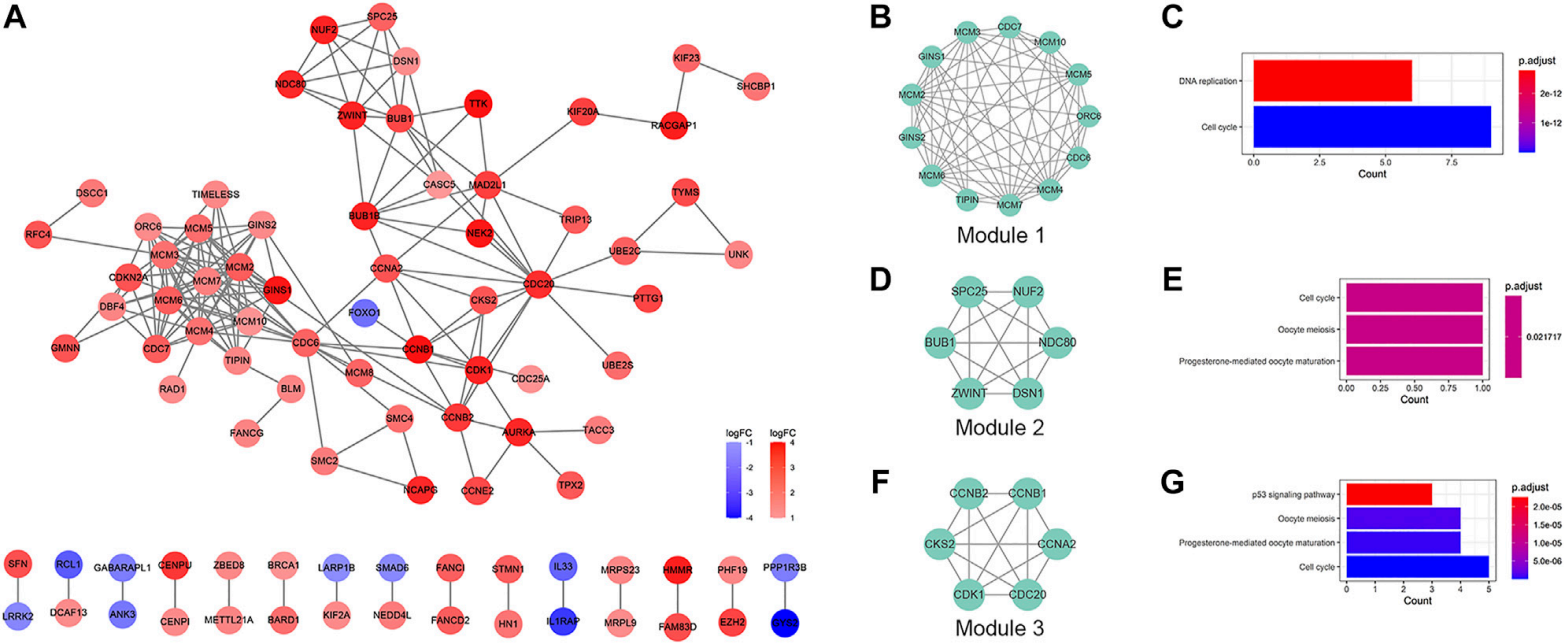
PPI network and module analysis. **(A)** PPI network of the DEGs. Red represents the upregulated DEGs, and blue represents the downregulated DEGs. **(B,D,F)** The selected modules of the PPI network. **(C,E,G)** KEGG pathway analysis of the modules. PPI, protein-protein interaction; DEGs, differentially expressed genes; KEGG, Kyoto Encyclopedia of Genes and Genomes.

**FIGURE 5:**
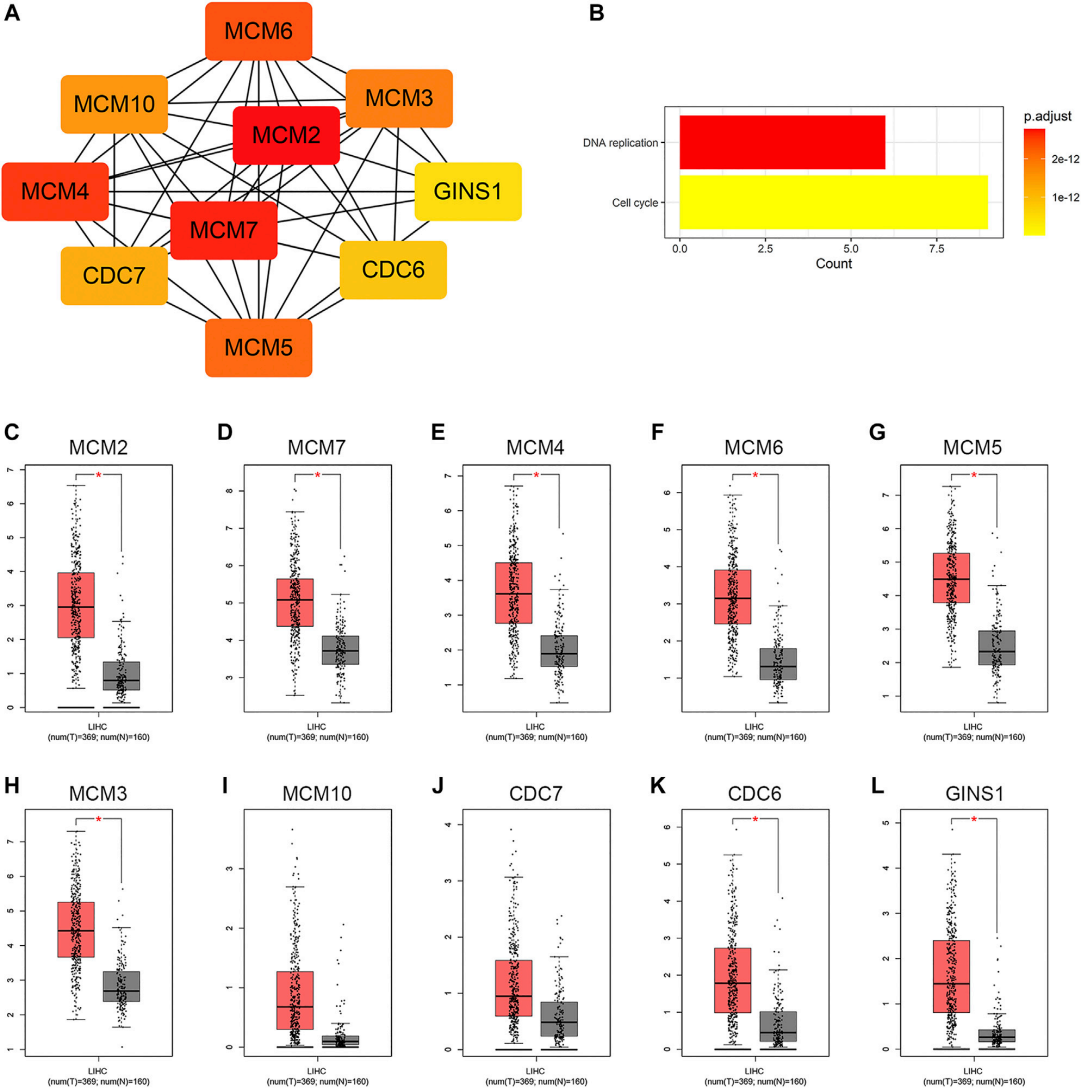
Identification of hub genes in the PPI network. **(A)** Hub gene network. **(B)** KEGG pathway analysis of the hub genes. **(C−L)** Differential expression analysis of the hub genes in GEPIA. Tumor tissue is shown in red, and normal tissue is shown in gray. PPI, protein-protein interaction; KEGG, Kyoto Encyclopedia of Genes and Genomes; GEPIA, Gene Expression Profiling Interactive Analysis; LIHC, liver hepatocellular carcinoma; T, tumor; N, normal.

Based on the STRING database and the Cytoscape software, a PPI network was constructed, consisting of 89 nodes and 178 edges (Figure 4A). As indicated in Figure 4A, the majority of the genes that met the filter settings in STRING database were upregulated. There were three modules selected after MCODE analysis (Figures 4B,D,F). With the highest score of 10.167, module 1 was significantly enriched in cell cycle and DNA replication (Figure 4C). Furthermore, module 2 was primarily associated with cell cycle, oocyte meiosis, and progesterone-mediated oocyte maturation (Figure 4E). Similarly, the most significant enrichment pathways of module 3 were cell cycle, oocyte meiosis, p53 signaling pathway, and progesterone-mediated oocyte maturation (Figure 4G). As important components of module 1, the ten genes (MCM2, MCM7, MCM4, MCM6, MCM5, MCM3, MCM10, CDC7, CDC6, and GINS1) were also ranked as hub genes of the whole network, enriched in cell cycle and DNA replication (Table 1; Figures 5A,B). The GEPIA database was used to verify the expression of the hub genes. Eight of the hub genes (MCM2, MCM7, MCM4, MCM6, MCM5, MCM3, CDC6, and GINS1) were confirmed to have significant differential expression according to the results (Figures 5C–L).

**TABLE 1:** The top ten genes ranked as hub genes by the MCC algorithm.

Rank	Name	Score
1	MCM2	17570
2	MCM7	17544
3	MCM4	17521
4	MCM6	17281
5	MCM5	16584
6	MCM3	15985
7	MCM10	10104
8	CDC7	6024
9	CDC6	5777
10	GINS1	5760

### Identification of miRNAs, lncRNAs, and circRNAs, and Construction of a ceRNA Network

**FIGURE 6:**
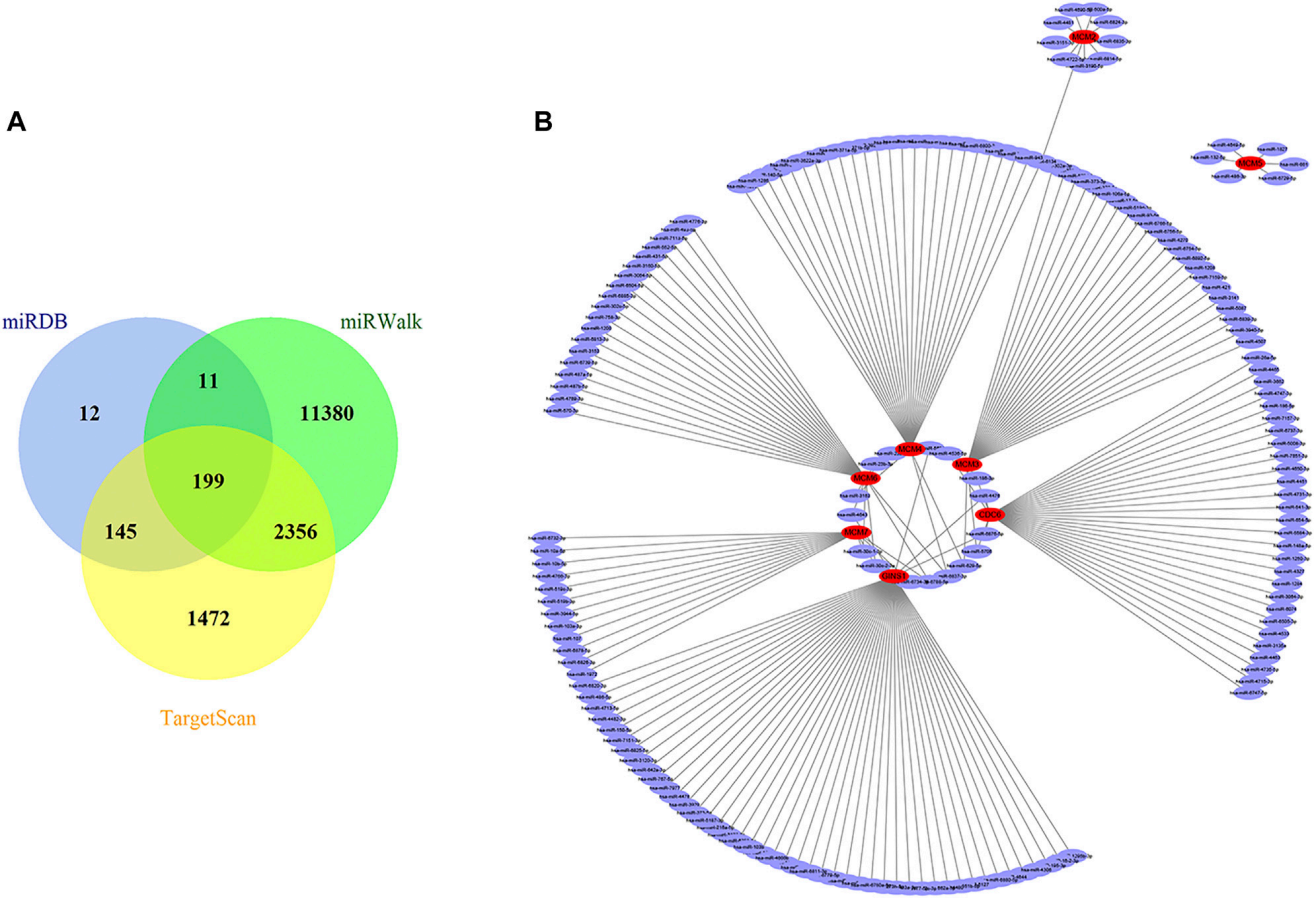
Prediction of targeted miRNAs. **(A)** Venn diagram of the common predicted miRNAs. **(B)** The miRNA-mRNA network constructed based on the predicted relationship. Red represents the mRNAs, and purple represents the miRNAs. MiRNAs, microRNAs; mRNAs, messenger RNAs.

A total of 199 overlapped upstream miRNAs related to the above eight key genes were predicted by searching miRDB, miRWalk, and TargetScan databases (Figure 6A). According to the corresponding relationship, a miRNA-mRNA network was established as shown in Figure 6B. The expression data of these predicted miRNAs were obtained in OncomiR database. It was found that only 15 of them had significant differential expression (Table 2).

**TABLE 2:** Differential expression analysis of the predicted miRNAs in OncomiR.

miRNA name	Cancer abbreviation	T-test *p*-value	Tumor Log2 mean expression	Normal Log2 mean expression
hsa-miR-1258	LIHC	4.44E-24	3.13E-21	0.59
hsa-miR-486-5p	LIHC	1.49E-12	6.08E-11	6.48
hsa-miR-30c-2-3p	LIHC	2.25E-10	3.87E-09	5.12
hsa-miR-214-5p	LIHC	7.94E-10	1.19E-08	2.4
hsa-miR-758-3p	LIHC	1.91E-09	2.37E-08	2.58
hsa-miR-10a-5p	LIHC	1.04E-08	1.02E-07	13.44
hsa-miR-30c-1-3p	LIHC	2.40E-08	2.11E-07	0.78
hsa-miR-195-3p	LIHC	2.17E-07	1.47E-06	1.49
hsa-miR-150-5p	LIHC	6.51E-07	4.10E-06	7.72
hsa-miR-26a-5p	LIHC	2.30E-06	1.25E-05	11.39
hsa-miR-23b-3p	LIHC	4.30E-05	1.74E-04	10.52
hsa-miR-19b-3p	LIHC	7.63E-03	1.81E-02	6.64
hsa-miR-148a-5p	LIHC	9.73E-03	2.24E-02	6.48
hsa-miR-651-5p	LIHC	2.89E-02	5.81E-02	1.68
hsa-miR-16-2-3p	LIHC	4.68E-02	8.54E-02	2.61

miRNAs, microRNAs; LIHC, liver hepatocellular carcinoma.

**FIGURE 7:**
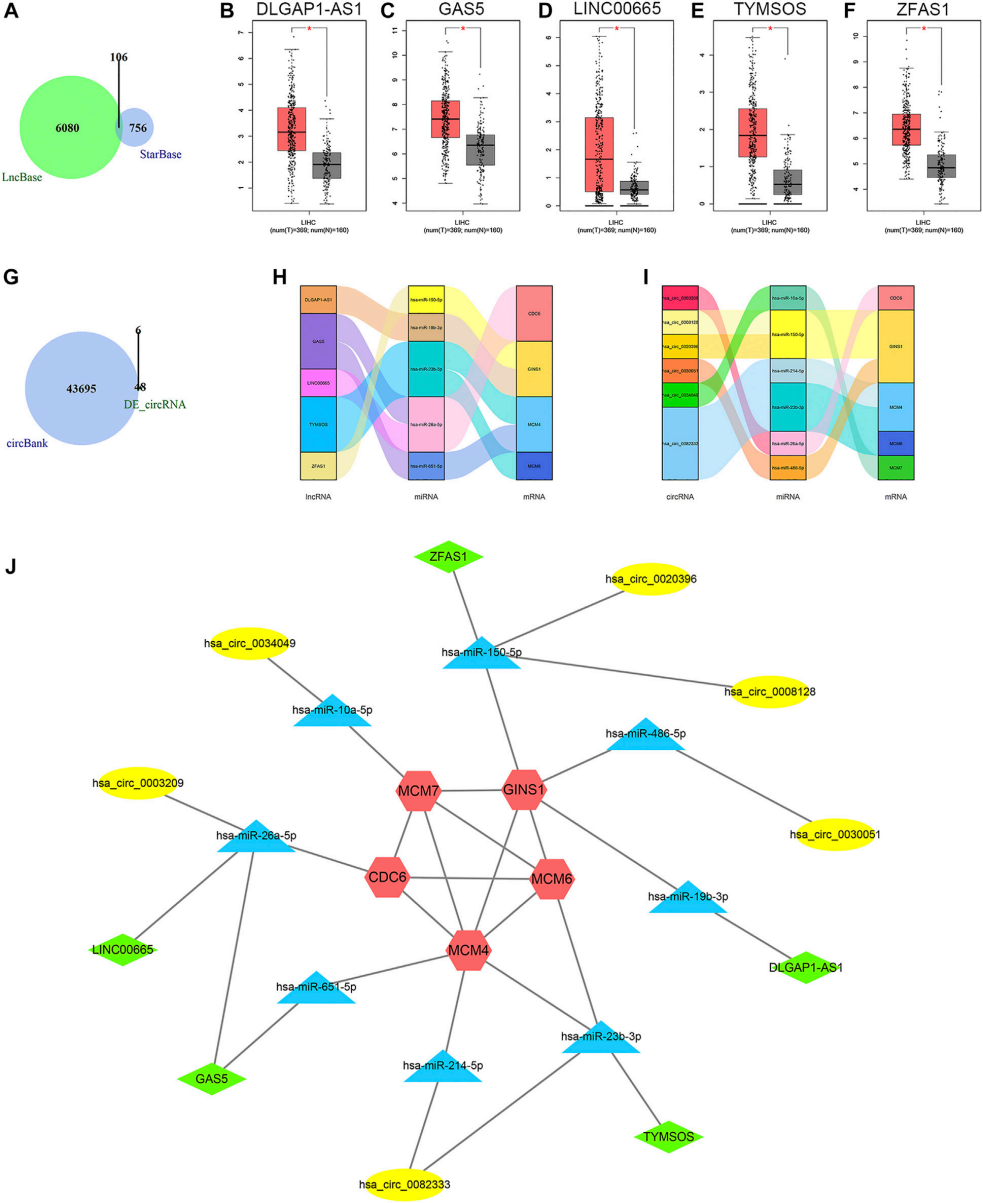
Results of ceRNA prediction and ceRNA network construction. **(A)** Venn diagram of the common predicted lncRNAs. **(B−F)** Differential expression analysis of the lncRNAs in GEPIA. Tumor tissue is shown in red, and normal tissue is shown in gray. **(G)** Venn diagram of the predicted circRNAs and differentially expressed circRNAs. Sankey diagrams of the lncRNA-miRNA-mRNA regulatory relationship **(H)** and circRNA-miRNA-mRNA regulatory relationship **(I)**. **(J)** The ceRNA network. Red represents the mRNAs, blue represents the miRNAs, green represents the lncRNAs, and yellow represents the circRNAs. CeRNA, competing endogenous RNA; lncRNA, long non-coding RNA; GEPIA, Gene Expression Profiling Interactive Analysis; circRNA, circular RNA; miRNA, microRNA; mRNA, messenger RNA; LIHC, liver hepatocellular carcinoma; T, tumor; N, normal; DE_circRNA, differentially expressed circular RNA.

On the basis of the aforementioned miRNAs, 106 common lncRNA-miRNA regulatory pairs were obtained using StarBase and LncBase (Figure 7A). Five eligible lncRNAs (DLGAP1-AS1, GAS5, LINC00665, TYMSOS, and ZFAS1) were screened out after the integration with the expression data from GEPIA (Figures 7B–F), corresponding to five miRNAs (hsa-miR-150-5p, hsa-miR-19b-3p, hsa-miR-23b-3p, hsa-miR-26a-5p, and hsa-miR-651-5p) and four mRNAs (CDC6, GINS1, MCM4, and MCM6). The circBank database revealed 43701 circRNA-mRNA interactions. Then we selected six circRNAs (hsa_circ_0003209, hsa_circ_0008128, hsa_circ_0020396, hsa_circ_0030051, hsa_circ_0034049, and hsa_circ_0082333) by integrating the microarray data from the GSE97332 dataset and the GSE164803 dataset (Figure 7G), corresponding to six miRNAs (hsa-miR-10a-5p, hsa-miR-150-5p, hsa-miR-214-5p, hsa-miR-23b-3p, hsa-miR-26a-5p, and hsa-miR-486-5p) and five mRNAs (CDC6, GINS1, MCM4, MCM6, and MCM7). The lncRNA/circRNA-miRNA-mRNA regulatory relationships were shown as the Sankey diagrams in Figure 7H and Figure 7I. A total of five lncRNAs, six circRNAs, eight miRNAs, and five mRNAs constituted the ceRNA network (Figure 7J).

### Diagnostic and Prognostic Value of Key Genes in Hepatocellular Carcinoma

**FIGURE 8:**
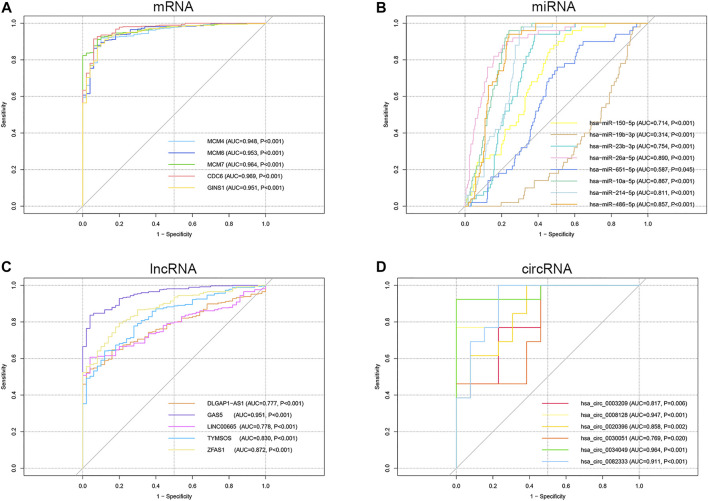
ROC curve analysis of the mRNA **(A)**, miRNA **(B)**, lncRNA **(C)**, and circRNA **(D)** in the ceRNA network. ROC, receiver operating characteristic; mRNA, messenger RNA; miRNA, microRNA; lncRNA, long non-coding RNA; circRNA, circular RNA; ceRNA, competing endogenous RNA; AUC, area under the ROC curve.

**FIGURE 9:**
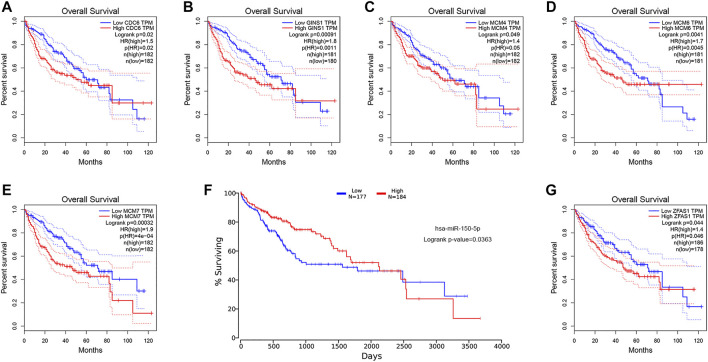
KM survival analysis of the mRNA **(A−E)**, miRNA hsa-miR-150-5p **(F)**, and lncRNA ZFAS1 **(G)**. KM, Kaplan-Meier; mRNA, messenger RNA; miRNA, microRNA; lncRNA, long non-coding RNA; TPM, transcripts per million; HR, hazard ratio.

ROC curve analysis was performed on the key genes in the ceRNA network (Supplementary Tables S2–S5). The results indicated that all the mRNAs, lncRNAs, circRNAs, and six of the miRNAs had good diagnostic value (AUC>0.7, *p* < 0.05), excluding two miRNAs hsa-miR-19b-3p (AUC = 0.314, *p* < 0.001) and hsa-miR-651-5p (AUC = 0.587, *p* = 0.045) (Figures 8A–D). Kaplan-Meier survival analysis demonstrated that the expression levels of all the mRNAs, one miRNA (hsa-miR-150-5p), and one lncRNA (ZFAS1) were significantly correlated with poor prognosis in HCC patients (*p* < 0.05) (Figures 9A–G). It was noticed that ZFAS1, hsa-miR-150-5p, and GINS1 formed a lncRNA-miRNA-mRNA axis that carried both diagnostic and prognostic significance.

### Gene Set Enrichment Analysis Analysis of GINS1 and Construction of a Conceptual Map

**FIGURE 10:**
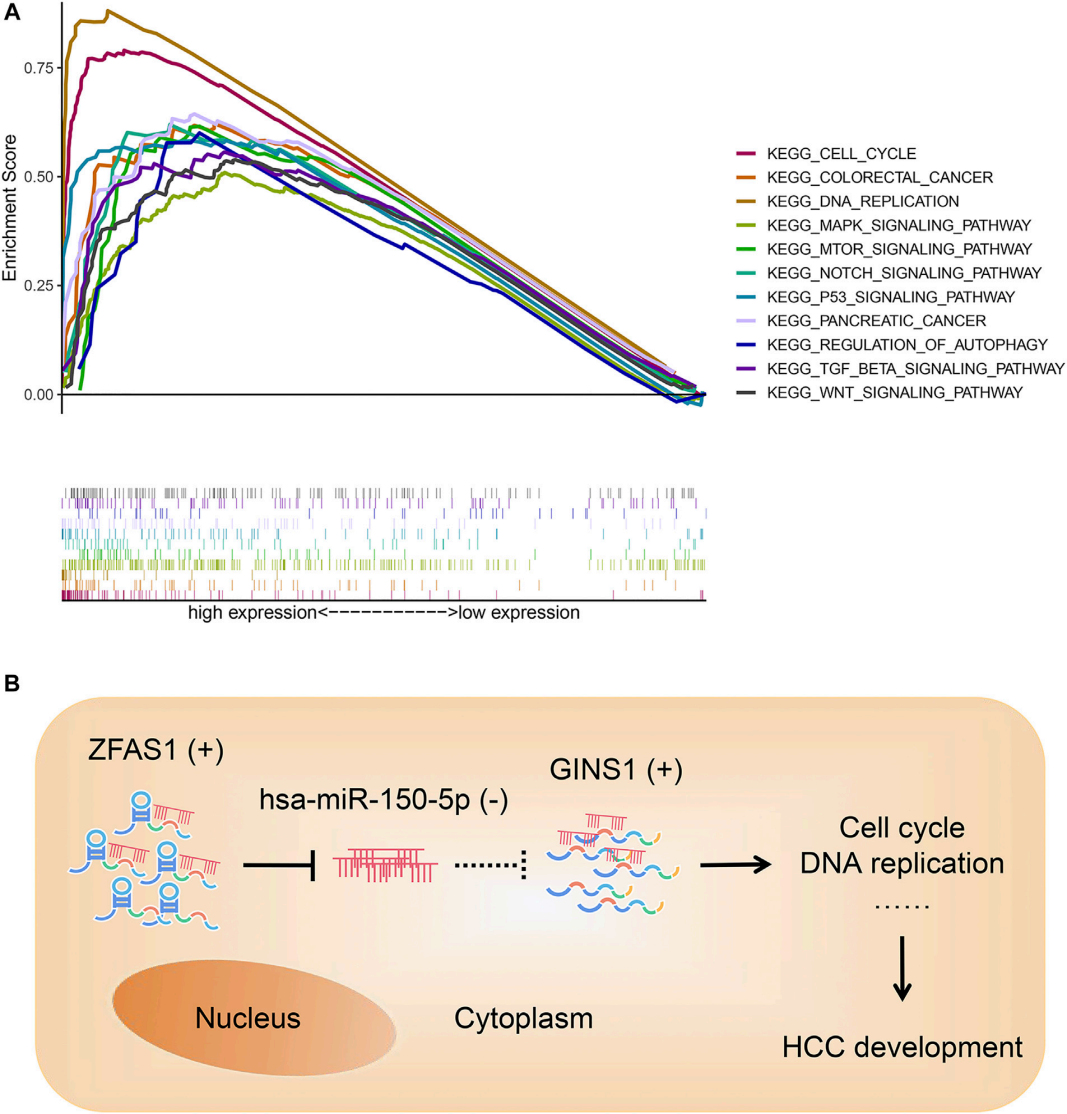
GSEA analysis of GINS1 **(A)** and conceptual map of the mechanism of ZFAS1/hsa-miR-150-5p/GINS1 axis **(B)**. In the cytoplasm of HCC cell, lncRNA ZFAS1 could act as a sponge to bind and negatively regulate the expression of miRNA hsa-miR-150-5p. Then miR-150-5p-mediated suppression of target mRNA was relieved and GINS1 continued to exert an oncogenic role in the development of HCC. GSEA, gene set enrichment analysis; HCC, hepatocellular carcinoma; lncRNA, long non-coding RNA; miRNA, microRNA; mRNA, messenger RNA; KEGG, Kyoto Encyclopedia of Genes and Genomes; (+), upregulated; (−), downregulated.

GSEA analysis showed that the core gene GINS1 was remarkably related to pathways contributing to HCC development and progression, such as cell cycle, DNA replication, p53 signaling pathway, mTOR signaling pathway, Notch signaling pathway, Wnt signaling pathway, and so on (Figure 10A). Based on our results, lncRNA ZFAS1 could sponge hsa-miR-150-5p and upregulate the expression of GINS1 in the cytoplasm of HCC cell. Shown in Figure 10B was our final conceptual map. The ZFAS1/hsa-miR-150-5p/GINS1 axis might directly or indirectly impact the HCC development through the pathways in Figure 10A.

## Discussion

Amounting evidence has shown that ceRNA might play a role in cancer initiation and progression (Qi et al., 2015). In the ceRNA hypothesis (Salmena et al., 2011), the ability of ceRNA to competitively bind to miRNA can influence tumorigenesis and cancer progression *via* regulating mRNA expression. To date, several ceRNAs have been identified to have a role in HCC (Xu G et al., 2020). However, there are still many ceRNAs of potential significance that have yet to be identified and require further exploration. Through bioinformatics analysis, this study attempted to establish a lncRNA/circRNA-miRNA-mRNA network holding biological functions in HCC. Integration of various databases would help achieve more reliable results. Based on the ceRNA hypothesis, a ceRNA network was successfully constructed *via* stepwise reverse prediction from mRNA to lncRNA/circRNA. Our results are expected to provide valuable guidance for HCC management.

The minichromosome maintenance (MCM) family is mainly known for their involvement in DNA replication (Maiorano et al., 2006). Given that DNA replication is a crucial pathway in tumor development, members of the MCM family are implied to be closely related to cancer development as well (Neves and Kwok, 2017; Yu et al., 2020). The overexpression of MCMs in various cancer tissues has been demonstrated by multiple studies, and is generally connected with poor prognostic features (Giaginis et al., 2011; Peng et al., 2016; Liu et al., 2017; Issac et al., 2019). Through functional enrichment analysis, we found that the hub genes with MCMs predominating were mainly involved in cell cycle and DNA replication pathways. There is evidence suggesting that high expression of MCM4 is correlated with clinicopathological variables and prognosis of HCC and silencing MCM4 can suppress the tumorigenicity of hepatoma cells (Xu et al., 2021). Liu et al. found that knockdown of MCM6 in Huh7 cells could cause a delay in S/G2-phase progression through down-regulating the cell cycle checkpoint (Liu et al., 2018). In addition, it has been demonstrated that MCM7 promotes cancer progression through cyclin D1-dependent signaling (Qu et al., 2017). The above studies have displayed the potential of MCMs as biomarkers to engage in HCC management, which is consistent with the results observed in this study. Known as a molecular switch, CDC6 is considered to have a transcriptional effect on E-Cadherin and subsequently affect EMT (Sideridou et al., 2011; Petrakis et al., 2012). In cancer cells, aberrant expression of CDC6 is involved in proliferation and tumor growth by modulating cell cycle (Lim and Townsend, 2020). Xu et al. revealed that CDC6 was regulated by miR-215-5p to involve in the proliferation of HCC (Xu H et al., 2020). They also found that CDC6 was negatively associated with overall and disease-free survival in HCC patients. In this study, CDC6 was modified by hsa-miR-26a-5p, which has been reported to have an effect on cell proliferation, migration, and invasion in digestive malignancies (Tian et al., 2019; Li H. H et al., 2020; Li Y et al., 2020; Zhang et al., 2020). The GINS complex, a component of the DNA replication machinery, usually participates in DNA replication through interactions with MCMs and other components (Labib and Gambus, 2007; Yoshimochi et al., 2008). GINS1 is a subunit of the GINS complex. Previous studies have demonstrated that GINS1 is upregulated in tumor samples and correlated with poor prognosis (Bu et al., 2020a; Bu et al., 2020b; Li H et al., 2021). These studies are consistent with our findings. The five mRNAs (CDC6, GINS1, MCM4, MCM6, and MCM7) in the ceRNA network were upregulated in HCC tissues and of great value in HCC diagnosis and prognosis.

MicroRNA (miRNA), a type of single-stranded nonencoding RNA, is widely involved in tumor generation and development (Krol et al., 2010). MiRNAs regulate gene expression primarily by degrading mRNA or inhibiting its translation (Bartel, 2009). In this ceRNA network, eight miRNAs (hsa-miR-150-5p, hsa-miR-19b-3p, hsa-miR-23b-3p, hsa-miR-26a-5p, hsa-miR-651-5p, hsa-miR-10a-5p, hsa-miR-214-5p and hsa-miR-486-5p) were found underexpressed in HCC samples. They linked the ceRNAs to the target mRNAs. Several miRNAs of them have been reported to have a place in proliferation, migration, and invasion of HCC cells (Li T. et al., 2014; Pang et al., 2018; He et al., 2019; Wu et al., 2019; Zhu et al., 2020; Hayashi et al., 2021). Similarly, there have been researches that explored the functions of the five lncRNAs (DLGAP1-AS1, GAS5, LINC00665, TYMSOS, and ZFAS1) on tumorigenesis and progression of HCC or other tumors (Zhang et al., 2019; Zhou et al., 2019; Ding et al., 2020; Lin et al., 2020; Gu et al., 2021). Their expression levels were highly relevant to tumor growth, invasion, and metastasis. With the development of high-throughput sequencing and emergence of bioinformatic methods, studies have increasingly revealed the important roles of circRNAs in various tumors (Lei et al., 2020; Li R et al., 2020). Their unique covalent closed-loop structures make them advantageous for clinical application. Due to the short development period, abundant circRNAs and their functions in HCC need further excavation. Our study reversely predicted six circRNAs (hsa_circ_0003209, hsa_circ_0008128, hsa_circ_0020396, hsa_circ_0030051, hsa_circ_0034049, and hsa_circ_0082333) and all of them had reliable diagnostic value in HCC.

Notably, our study discovered a lncRNA-miRNA-mRNA axis of great clinical significance, namely ZFAS1/hsa-miR-150-5p/GINS1 ceRNA axis. Their expression levels and predicted interactions in HCC are in line with the ceRNA hypothesis. LncRNA ZFAS1 may contribute to tumorigenesis of HCC by sponging hsa-miR-150-5p and regulating the expression of the target mRNA GINS1. Moreover, the diagnosis and prognosis analysis of each gene showed favourable outcomes (*p* < 0.05). Our GSEA analysis found that the target mRNA GINS1 was significantly associated with cell cycle, DNA replication, p53 signaling pathway, mTOR signaling pathway, Notch signaling pathway, Wnt signaling pathway, etc. The majority of these pathways have been confirmed to be involved in HCC development (Greenbaum, 2004; Giovannini et al., 2016; Dimri and Satyanarayana, 2020). The previous study revealed that ZFAS1 acted as an oncogene in HCC progression by binding miR-150 and abrogating its tumor-suppressive function (Li et al., 2015). Besides, *in vitro* experiments have verified that miR-150-5p inhibition significantly promotes hepatoma cell migration and invasion (Li T et al., 2014). GINS1 was reported to be associated with tumor grades and poor survival of HCC patients (Li S et al., 2021). Furthermore, cell cycle, cell proliferation assay, and *in vivo* animal model experiment indicated that knocking down GINS1 induced in G1/S phase cell cycle arrest and decreased HCC cells proliferation (Li S et al., 2021). These studies provide excellent support for our studies.

## Conclusion

In summary, through integrating data from a variety of databases, we successfully constructed a ceRNA network containing five lncRNAs, six circRNAs, eight miRNAs, and five mRNAs. The ceRNA network can promote HCC progression *via* cell cycle, DNA replication, and other pathways. Clinical diagnostic and survival analyses demonstrated that the ZFAS1/hsa-miR-150-5p/GINS1 ceRNA regulatory axis had a high diagnostic and prognostic value. Our findings are expected to provide potential biomarkers and therapeutic targets for HCC management. Nevertheless, our study also presents some limitations. Results in this study are based on bioinformatic predictions and further experiments and clinical practice are warranted. Additionally, since circRNAs has not been fully studied in HCC, data of circRNAs from the available datasets are limited and insufficient for prognostic analysis. We expect more research with larger sample sizes to expand and refine our conclusions.

## Data Availability

Publicly available datasets were analyzed in this study. This data can be found here: GEO database (https://www.ncbi.nlm.nih.gov/geo/) and TCGA database (https://portal.gdc.cancer.gov/).

## Footnotes

1
https://www.ncbi.nlm.nih.gov/geo/

2
http://www.webgestalt.org

3
https://string-db.org/

4
http://gepia.cancer-pku.cn/

5
http://mirdb.org/

6
http://mirwalk.umm.uni-heidelberg.de/

7
http://www.targetscan.org/vert_72/

8
http://www.oncomir.org/

9
http://starbase.sysu.edu.cn/

10
http://carolina.imis.athena-innovation.gr/diana_tools/web/index.php?r=lncbasev2/index-predicted

11
http://www.circbank.cn/

12
https://portal.gdc.cancer.gov/

13
http://www.oncolnc.org/
