# Supplementary material for: Identification of lncRNA/circRNA-miRNA-mRNA ceRNA Network as Biomarkers for Hepatocellular Carcinoma
Source: Front Genet. 2022 Mar 21;13:838869. doi: 10.3389/fgene.2022.838869 (PMC8977626; doi:10.3389/fgene.2022.838869)
Supplement: Supplementary file 1 [file Table1.DOCX]

**Supplementary Table S1**. GO and KEGG analysis of DEGs in hepatocellular carcinoma.

| Expression | Category | GeneSet | Description | EnrichmentRatio | *P*-Value | FDR |
| --- | --- | --- | --- | --- | --- | --- |
| Upregulated | BP | GO:0007049 | cell cycle | 4.824531979 | 0 | 0 |
|  |  | GO:0022402 | cell cycle process | 5.813361242 | 0 | 0 |
|  |  | GO:0051726 | regulation of cell cycle | 4.603777376 | 0 | 0 |
|  |  | GO:0006259 | DNA metabolic process | 4.593098511 | 0 | 0 |
|  |  | GO:0000278 | mitotic cell cycle | 6.990770706 | 0 | 0 |
|  |  | GO:0006974 | cellular response to DNA damage stimulus | 3.804762889 | 0 | 0 |
|  |  | GO:0007346 | regulation of mitotic cell cycle | 5.906237985 | 0 | 0 |
|  |  | GO:0045786 | negative regulation of cell cycle | 5.382428941 | 0 | 0 |
|  |  | GO:0000226 | microtubule cytoskeleton organization | 4.607458848 | 0 | 0 |
|  |  | GO:0045930 | negative regulation of mitotic cell cycle | 7.232638889 | 0 | 0 |
|  | CC | GO:0044430 | cytoskeletal part | 3.473401809 | 0 | 0 |
|  |  | GO:0015630 | microtubule cytoskeleton | 4.650541898 | 0 | 0 |
|  |  | GO:0005694 | chromosome | 7.024331425 | 0 | 0 |
|  |  | GO:0044427 | chromosomal part | 6.833263268 | 0 | 0 |
|  |  | GO:0005815 | microtubule organizing center | 4.643073329 | 0 | 0 |
|  |  | GO:0000228 | nuclear chromosome | 6.71246582 | 0 | 0 |
|  |  | GO:0044454 | nuclear chromosome part | 6.257300692 | 0 | 0 |
|  |  | GO:0098687 | chromosomal region | 12.05044216 | 0 | 0 |
|  |  | GO:0005819 | spindle | 10.2062679 | 0 | 0 |
|  |  | GO:0000793 | condensed chromosome | 14.05370273 | 0 | 0 |
|  | MF | GO:0005524 | ATP binding | 2.544248984 | 3.10E-11 | 2.95E-08 |
|  |  | GO:0015631 | tubulin binding | 5.051092767 | 3.14E-11 | 2.95E-08 |
|  |  | GO:0032559 | adenyl ribonucleotide binding | 2.449830202 | 1.36E-10 | 6.50E-08 |
|  |  | GO:0008017 | microtubule binding | 5.698647087 | 1.38E-10 | 6.50E-08 |
|  |  | GO:0030554 | adenyl nucleotide binding | 2.428905239 | 1.89E-10 | 7.08E-08 |
|  |  | GO:0140097 | catalytic activity, acting on DNA | 6.344611741 | 5.29E-10 | 1.65E-07 |
|  |  | GO:0008144 | drug binding | 2.24165544 | 1.88E-09 | 5.03E-07 |
|  |  | GO:0035639 | purine ribonucleoside triphosphate binding | 2.178814036 | 3.89E-09 | 9.13E-07 |
|  |  | GO:0032555 | purine ribonucleotide binding | 2.103438847 | 1.45E-08 | 3.03E-06 |
|  |  | GO:0016887 | ATPase activity | 3.701828261 | 1.95E-08 | 3.06E-06 |
|  | KEGG | hsa04110 | Cell cycle | 12.9770624 | 0 | 0 |
|  |  | hsa03030 | DNA replication | 18.2106102 | 7.93E-12 | 1.29E-09 |
|  |  | hsa04115 | p53 signaling pathway | 8.277550091 | 2.72E-07 | 2.96E-05 |
|  |  | hsa03460 | Fanconi anemia pathway | 8.829386764 | 2.72E-06 | 2.22E-04 |
|  |  | hsa04914 | Progesterone-mediated oocyte maturation | 5.418032787 | 3.82E-05 | 0.001851837 |
|  |  | hsa05206 | MicroRNAs in cancer | 4.370546448 | 3.92E-05 | 0.001851837 |
|  |  | hsa04114 | Oocyte meiosis | 4.806319408 | 3.98E-05 | 0.001851837 |
|  |  | hsa04218 | Cellular senescence | 4.097387295 | 7.10E-05 | 0.002892648 |
|  |  | hsa00240 | Pyrimidine metabolism | 4.72066223 | 2.74E-04 | 0.009928488 |
|  |  | hsa03440 | Homologous recombination | 7.268092763 | 5.66E-04 | 0.01845693 |
| Downregulated | BP | GO:0044281 | small molecule metabolic process | 2.771383471 | 0 | 0 |
|  |  | GO:0006082 | organic acid metabolic process | 3.534380367 | 0 | 0 |
|  |  | GO:0043436 | oxoacid metabolic process | 3.557905613 | 0 | 0 |
|  |  | GO:0055114 | oxidation-reduction process | 3.328843432 | 0 | 0 |
|  |  | GO:0019752 | carboxylic acid metabolic process | 3.788144567 | 0 | 0 |
|  |  | GO:0032787 | monocarboxylic acid metabolic process | 4.162997838 | 0 | 0 |
|  |  | GO:0016054 | organic acid catabolic process | 6.102879625 | 0 | 0 |
|  |  | GO:0046395 | carboxylic acid catabolic process | 6.102879625 | 0 | 0 |
|  |  | GO:0006954 | inflammatory response | 3.531134176 | 2.22E-16 | 2.24E-13 |
|  |  | GO:0017144 | drug metabolic process | 3.331346322 | 2.78E-15 | 2.52E-12 |
|  | CC | GO:0031226 | intrinsic component of plasma membrane | 2.332739112 | 1.17E-10 | 6.97E-08 |
|  |  | GO:0005887 | integral component of plasma membrane | 2.367655494 | 1.37E-10 | 6.97E-08 |
|  |  | GO:0005783 | endoplasmic reticulum | 2.23023174 | 1.78E-10 | 6.97E-08 |
|  |  | GO:0009986 | cell surface | 3.010221174 | 1.27E-09 | 3.73E-07 |
|  |  | GO:0005759 | mitochondrial matrix | 3.486205109 | 3.56E-08 | 8.35E-06 |
|  |  | GO:0044432 | endoplasmic reticulum part | 2.185824028 | 2.91E-07 | 5.69E-05 |
|  |  | GO:0009897 | external side of plasma membrane | 3.858302062 | 1.15E-06 | 1.94E-04 |
|  |  | GO:0044429 | mitochondrial part | 2.258173177 | 2.98E-06 | 4.38E-04 |
|  |  | GO:0098552 | side of membrane | 2.962131416 | 3.71E-06 | 4.84E-04 |
|  |  | GO:0042175 | nuclear outer membrane-endoplasmic reticulum membrane network | 2.138102402 | 1.03E-05 | 0.001195317 |
|  | MF | GO:0016491 | oxidoreductase activity | 4.200302398 | 0 | 0 |
|  |  | GO:0048037 | cofactor binding | 4.853872486 | 0 | 0 |
|  |  | GO:0050662 | coenzyme binding | 5.319866568 | 2.90E-14 | 1.81E-11 |
|  |  | GO:0004497 | monooxygenase activity | 9.23434903 | 7.05E-13 | 3.31E-10 |
|  |  | GO:0005506 | iron ion binding | 6.586059744 | 2.53E-11 | 7.91E-09 |
|  |  | GO:0020037 | heme binding | 6.853618421 | 1.30E-10 | 3.49E-08 |
|  |  | GO:0046906 | tetrapyrrole binding | 6.356979405 | 4.61E-10 | 1.08E-07 |
|  |  | GO:0033218 | amide binding | 3.72774713 | 4.66E-09 | 9.71E-07 |
|  | KEGG | hsa00071 | Fatty acid degradation | 9.676801802 | 3.17E-10 | 1.03E-07 |
|  |  | hsa00380 | Tryptophan metabolism | 9.825675676 | 1.28E-09 | 2.09E-07 |
|  |  | hsa01100 | Metabolic pathways | 1.882313348 | 6.53E-09 | 7.10E-07 |
|  |  | hsa00830 | Retinol metabolism | 6.354914616 | 8.32E-08 | 6.78E-06 |
|  |  | hsa00232 | Caffeine metabolism | 26.2018018 | 4.13E-06 | 2.69E-04 |
|  |  | hsa05204 | Chemical carcinogenesis | 4.793012525 | 6.12E-06 | 3.32E-04 |
|  |  | hsa00350 | Tyrosine metabolism | 7.278278278 | 9.63E-06 | 4.48E-04 |
|  |  | hsa00982 | Drug metabolism | 4.548923924 | 5.87E-05 | 0.002392301 |
|  |  | hsa00280 | Valine, leucine and isoleucine degradation | 5.458708709 | 8.75E-05 | 0.003062193 |
|  |  | hsa00980 | Metabolism of xenobiotics by cytochrome P450 | 4.309506875 | 9.39E-05 | 0.003062193 |

GO, Gene Ontology; KEGG, Kyoto Encyclopedia of Genes and Genomes; DEGs, differentially expressed genes; BP, biological process; CC, cellular component, MF, molecular function; FDR, false discovery rate.
